# Associations of sense of belonging and academic self-efficacy with college persistence through student engagement: MASEM with moderation by learning environment

**DOI:** 10.3389/fpsyg.2026.1858552

**Published:** 2026-09-30

**Authors:** Fatma Uslu Gülşen

**Affiliations:** Department of Educational Administration, Faculty of Education, Mersin University, Mersin, Türkiye

**Keywords:** sense of belonging, academic self-efficacy, engagement, MASEM, persistence

## Abstract

Previous research, including meta-analytic evidence, has established positive associations among sense of belonging, academic self-efficacy, engagement, and persistence. However, it remains unclear how these factors interact through student engagement and whether these associations differ across learning environments. This study applied meta-analytic structural equation modelling (MASEM) to synthesize findings from 20 independent studies and examine the direct and indirect associations among these variables. A partial mediation model was initially tested, in which sense of belonging and academic self-efficacy were specified as both directly and indirectly associated with persistence through engagement. Although the initial partial mediation model demonstrated acceptable fit, the direct association between academic self-efficacy and persistence was not statistically significant. Following this analysis, a revised partial mediation model was examined as a post-hoc exploratory specification and showed favorable fit indices, with both academic self-efficacy and sense of belonging significantly associated with engagement, which, in turn, was positively associated with persistence. Notably, sense of belonging also retained a significant direct association with persistence, whereas academic self-efficacy was related to persistence primarily through its association with engagement. A full mediation model was also examined and showed less favorable fit indices. Moderator analyses indicated that the associations involving sense of belonging varied across learning environments, whereas the other examined paths did not significantly differ by learning environment. However, sensitivity analysis indicated limited stability for the sense of belonging–engagement pathway in the online subgroup. Overall, the findings highlight the central role of engagement in understanding student persistence. Given the predominantly observational nature of the included studies, the indirect effects should be interpreted as statistical associations rather than evidence of causal mediation.

## 1 Introduction

College persistence is widely recognized as one of the most important indicators of participation in higher education. Understanding why some students remain enrolled while others withdraw has therefore become a central concern in the field. Much of this work has been guided by Tinto’s (1975) Model of Student Departure, which highlights how students’ initial aspirations interact with their academic and social integration into college.

Within this perspective, engagement, which refers to students’ active involvement in academic activities and institutional life (Kuh, 2001), has emerged as a key mechanism connecting students’ experiences to persistence. A body of research shows that students who invest more time and effort in educational activities are more likely to remain enrolled and progress toward degree completion (Kuh et al., 2008). At the same time, engagement is often understood as a behavioural pathway through which various psychosocial factors influence academic outcomes (Kahu and Nelson, 2018).

Among these psychosocial factors, sense of belonging and academic self-efficacy are particularly prominent. Sense of belonging refers to students’ perceived inclusion and acceptance within the academic community (Goodenow, 1993), whereas academic self-efficacy refers to their beliefs in their ability to successfully perform academic tasks (Bandura, 1997). Research indicates that students who feel a stronger sense of belonging and possess higher academic self-efficacy tend to be more engaged and more likely to persist (Gillen-O’Neel, 2019; Zumbrunn et al., 2014).

Recent developments in higher education have further complicated this situation. According to Muljana and Luo (2019) the rapid expansion of online learning has introduced new challenges related to engagement and integration. While online environments offer flexibility and increased access (Allen and Seaman, 2017), they may also weaken related psychosocial factors. As a result, the associations among variables of this study may not operate in the same way across different learning environments.

Although studies report that higher engagement is associated with stronger persistence (Jung and Lee, 2018; Lee and Song, 2022), evidence on how sense of belonging, academic self-efficacy, engagement, and persistence are structurally related remains fragmented. Previous meta-analytic studies have examined specific parts of these relationships, including belonging in relation to academic success and persistence (Moallem, 2013), academic self-efficacy in relation to engagement (Chang and Chien, 2015), and social-cognitive predictors of academic performance and persistence (Brown et al., 2008). Individual studies have also reported different patterns of direct and indirect associations with persistence (Hausmann et al., 2007; Jung and Lee, 2018; Lee and Song, 2022). In addition, there is limited evidence on whether these relationships differ between learning environments. Thus, this study employs MASEM to integrate these relationships within a single model, compare alternative model specifications, and examine whether the structural associations vary across face-to-face and online learning environments.

## 2 Theoretical framework

The study is grounded in Social Cognitive Theory, which emphasizes the dynamic interplay between personal beliefs, behavioral patterns, and environmental conditions (Bandura, 1997). It is also informed by Tinto’s integration model, which emphasizes academic and social integration as central to students’ persistence (Tinto, 1975, 1993). While SCT explains how self-efficacy is linked to behavioral involvement, Tinto’s model highlights how students’ sense of belonging and connection to the academic community are associated with engagement and persistence. These theoretical frameworks provide complementary explanations of the personal and social processes underlying persistence. In this context, SCT explains the interdependence among personal, environmental, and behavioral dimensions and thus posits that supportive learning opportunities foster academic self-efficacy and sense of belonging, and those in turn enhance engagement (Schunk and DiBenedetto, 2020). Engagement then serves as the behavioral pathway that sustains persistence (Eccles and Wigfield, 2020). From Tinto’s integration model, engagement also reflects students’ involvement in academic and social life, linking integration with persistence. Tinto’s model directly informs the present study by suggesting that sense of belonging and academic self-efficacy may be associated with persistence both directly and indirectly through engagement. Moreover, both theoretical frameworks recognize the importance of students’ interactions with their learning environments, and the strength of these associations may vary across learning environments, providing a basis for examining learning environment as a moderator.

## 3 Literature review

### 3.1 Persistence and engagement

College persistence refers to students’ continued enrolment and progress toward degree completion (Tinto, 1993). Research on college persistence has identified multiple theoretical models, most notably Astin’s involvement theory, Tinto’s student integration model, and Bean’s student attrition model. These models emphasize that persistence is shaped by the interaction between students’ academic experiences, social integration, and institutional environments (Astin, 1984; Bean, 1980; Tinto, 1975). In particular, student integration model (Tinto, 1975) suggests that students who become socially and academically integrated into the institution are more likely to persist. Building on this, Dolan (2019) shows that persistence is best understood as a multifaceted process, where integration, commitment, and organizational factors jointly influence persistence.

Within these frameworks, engagement has emerged as a key mechanism linking students’ experiences to persistence. As conceptualized by Kuh (2001), engagement encompasses students’ dedication of effort and time to learning activities, along with the institution’s investment in high-impact educational practices, which are key factors determining academic success. Kuh et al. (2008) further demonstrated that engagement can act as a compensatory factor, supporting persistence particularly among first- and second-year students. Studies also indicate that engagement is shaped by instructional practices, learning environments, and digital tools (Bond et al., 2020; Li and Xue, 2023).

Research highlights the central role of engagement in persistence. For instance, Lee and Song (2022) and Jung and Lee (2018) found that engagement significantly predicts persistence in Massive Online Open Course (MOOC) contexts. Similarly, engagement is often positioned as a mediator linking students’ individual characteristics to academic outcomes (Alamri, 2022; Papa, 2015), making differences in model specification an important source of variation across findings. These variations likely reflect differences in learning contexts, construct operationalization, and model specification, particularly whether engagement is treated as a broad construct or differentiated into behavioral, cognitive, and social dimensions. Consequently, differences in effect sizes may reflect methodological and conceptual heterogeneity rather than genuinely contradictory evidence.

In addition, Hu (2011) found that high academic engagement without sufficient social engagement may reduce persistence, whereas active social participation supports it. Findings regarding the antecedents of engagement are mixed. Osterman (2000) demonstrates that students who feel a greater sense of belonging exhibit higher levels of engagement. However, meta-analytic findings are inconsistent, with sense of belonging showing a small effect on engagement (Moallem, 2013), academic self-efficacy demonstrating a moderate effect (Chang and Chien, 2015), and others reporting a large effect on engagement (Myint and Khaing, 2020). These variations likely reflect differences in research settings, definition and measurement of engagement, and the analytical strategies employed.

This emphasizes the importance of considering both academic and social dimensions of engagement in persistence models. Interventions aim at strengthening students’ sense of belonging may further illustrate this association. Practices such as peer support programs, community-building initiatives, and orientation activities have been shown to improve persistence (Gopalan and Brady, 2020; Murphy et al., 2020). These interventions may help students reinterpret academic challenges, foster meaningful relationships with peers and faculty, and ultimately strengthen their sense of belonging.

### 3.2 Sense of belonging and academic self-efficacy

Sense of belonging captures the extent to which individuals feel valued, supported, and connected to their institution (Goodenow, 1993). It plays a particularly important role during the transition to college, shaping students’ academic and social integration as well as their likelihood of persisting. Strayhorn (2019) conceptualizes sense of belonging as a universal human need, shaping students’ connection to peers, faculty, and institutions. Consistent with this, research shows that initiatives designed to enhance students’ sense of belonging significantly improve persistence outcomes (Gopalan and Brady, 2020; Lewis et al., 2017; Murphy et al., 2020). In addition, a growing body of evidence points to a positive association between students’ sense of belonging and their academic self-efficacy (Devlin and McKay, 2017).

Academic self-efficacy can be understood as students’ perceived capability to successfully handle academic challenges. Prior research has linked higher self-efficacy to increased persistence (Han et al., 2017; Sha, 2010), with effect sizes typically indicating modest to moderate associations across studies (Lewis et al., 2017; Lee and Song, 2022). In addition, several studies have documented a positive association between sense of belonging and academic self-efficacy (Zumbrunn et al., 2014; Won et al., 2019), as well as their joint associations with persistence (Gillen-O’Neel, 2019; Swanson et al., 2021). Both constructs have also been linked to student engagement; however, findings remain inconsistent, with some studies reporting strong positive associations (Vayre and Vonthron, 2019), while others have found weak or non-significant relationships (Jung and Lee, 2018; Wilson et al., 2015). Such variation may reflect differences in learning environments, construct measurement, and the way engagement is specified within structural models. Rather than indicating uniformly conflicting evidence, these differences raise the question of whether sense of belonging and academic self-efficacy are associated with persistence directly, indirectly through engagement, or through both pathways.

Meta-analytic evidence further supports a positive association between academic self-efficacy and persistence (Brown et al., 2008), although the strength and nature of this relationship vary across studies. Some research reports relatively strong associations (Lee and Song, 2022), whereas others find non-significant relationships (Jung and Lee, 2018). Such discrepancies may stem from differences in theoretical frameworks and model specifications. For example, Jung and Lee (2018) conceptualized engagement as the primary mediating mechanism, suggesting the association between academic self-efficacy and persistence operates indirectly through engagement. In contrast, Lee and Song (2022), drawing on expectancy–value theory, reported a direct association between academic self-efficacy and persistence, alongside a partial mediating role of engagement. These inconsistencies underscore the need for model comparison, testing alternative pathways to determine whether persistence is best explained by partial mediation, in which sense of belonging and academic self-efficacy exert both direct and indirect effects, or by full mediation, in which their association operates through engagement.

### 3.3 Moderation by learning environments

In traditional settings, opportunities for interpersonal communication and real-time feedback may foster sense of belonging and, in turn, persistence (Strayhorn, 2019). These environments may also provide increased access to peer interaction and faculty support, which are associated with higher levels of academic self-efficacy. In contrast, online learning environments often present additional challenges for persistence. Limited opportunities for direct interaction, combined with greater demands for self-regulation, can make it more difficult for students to feel connected and engaged (Muljana and Luo, 2019). As a result, students in online settings may experience lower levels of sense of belonging (Aversa and MacCall, 2013; Koole, 2014). At the same time, academic self-efficacy tends to play a more critical role in these contexts, as success in online learning depends heavily on students’ ability to manage their own learning processes. Supporting this view, Zimmerman and Kulikowich (2016) found that students with higher academic self-efficacy in online environments are more likely to persist. Prior research shows that the effects of academic self-efficacy vary across learning environments (Jung and Lee, 2018; Lewis et al., 2017). Studies also highlight contextual moderation, with sense of belonging exerting greater influence in face-to-face settings and academic self-efficacy more central in online environments (Muljana and Luo, 2019; Vayre and Vonthron, 2019; Zimmerman and Kulikowich, 2016; see Figures 1, 2).

**Figure 1:**
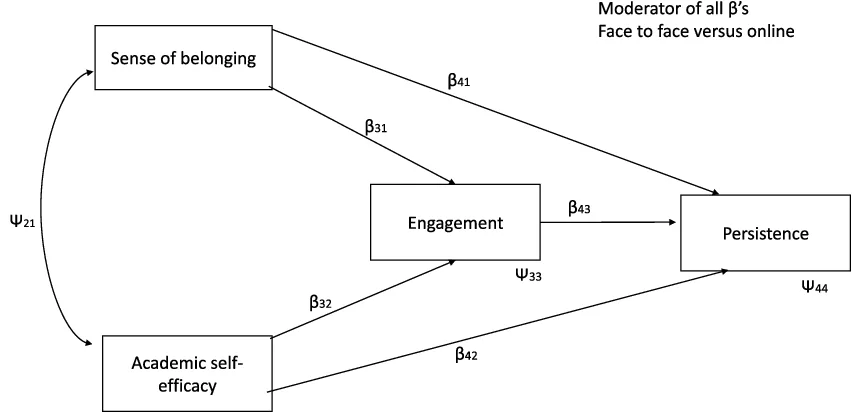
Partial mediation model.

**Figure 2:**
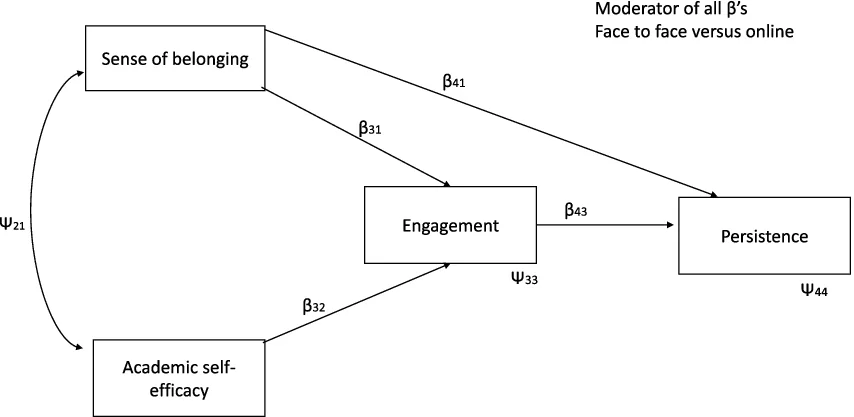
Full mediation model.

Based on this literature, the hypotheses tested in this study are as follows:

*H1:* The associations of sense of belonging and academic self-efficacy with persistence will be partially mediated by engagement.

*H2:* The associations of sense of belonging and academic self-efficacy with persistence will be fully mediated by engagement.

*H3:* The direct paths in the model will differ between face-to-face and online learning environments.

## 4 Methods

By extending structural equation modelling, MASEM integrates data from multiple studies through meta-analysis, enhancing the generalizability and robustness of findings across a broader evidence base. Since the variables are interrelated, I used multivariate MASEM to examine their associations with college persistence simultaneously within a structural framework. Correlation-based MASEM (Cheung and Chan, 2005) was used to synthesize the retrieved correlation matrices. In Stage 1, the available correlation matrices were synthesized using a random-effects model (Cheung, 2015a), and in Stage 2, the resulting pooled correlation structure was used to estimate the hypothesized structural models.

### 4.1 Study selection

I searched Google Scholar, Scopus, ERIC, PsycINFO, Web of Science databases using the following Boolean search string by following keywords: (“sense of belonging” OR belonging) AND (“academic self-efficacy” OR self-efficacy) AND (engagement OR “cognitive engagement” OR “behavioral engagement” OR “emotional engagement”) AND (persistence OR “intent to persist” OR retention). Broader terms (e.g., “self-efficacy,” “engagement”) were intentionally included to ensure comprehensive coverage of related constructs. Search terms were standardized (e.g., “sense of belonging” rather than “a sense of belonging”) to maximize retrieval. The literature search was performed between April and July 2025.

### 4.2 Screening, inclusion and exclusion

In the first stage of screening, studies were assessed according to their population, research design, publication language, and publication type. Studies were eligible if they (i) included college or university students, (ii) employed a quantitative research design, (iii) were written in English, and (iv) were published as journal articles or theses/dissertations. Of the 842 records initially identified, 220 duplicate records were removed, leaving 622 unique records for title and abstract screening. At this stage, 402 records were excluded because they were literature reviews, qualitative studies, studies published in languages other than English, or studies involving non-university student populations. This resulted in 220 reports proceeding to full-text eligibility assessment.

In the second step, I checked the accessibility and statistical reports of the studies. Full texts or additional sources providing sufficient information about the study had to be accessible. The correlation coefficients relevant to the study variables had to be reported or derivable from the available statistical information. For a study to be included in the analysis, at least three correlation coefficients had to be reported or derivable. To mitigate the possibility of duplication with existing research, I eliminated any dissertations that were subsequently published as a journal article. Of the 220 reports assessed for eligibility, 200 were excluded because they did not provide eligible data for the analysis (*n* = 105), did not report the required correlations (*n* = 47), or involved non-university student populations (*n* = 48). Consequently, 20 independent studies met the eligibility criteria and were included in the MASEM. The study selection process was reported in accordance with PRISMA 2020 (Page et al., 2021) and is presented in Figure 3.

**Figure 3:**
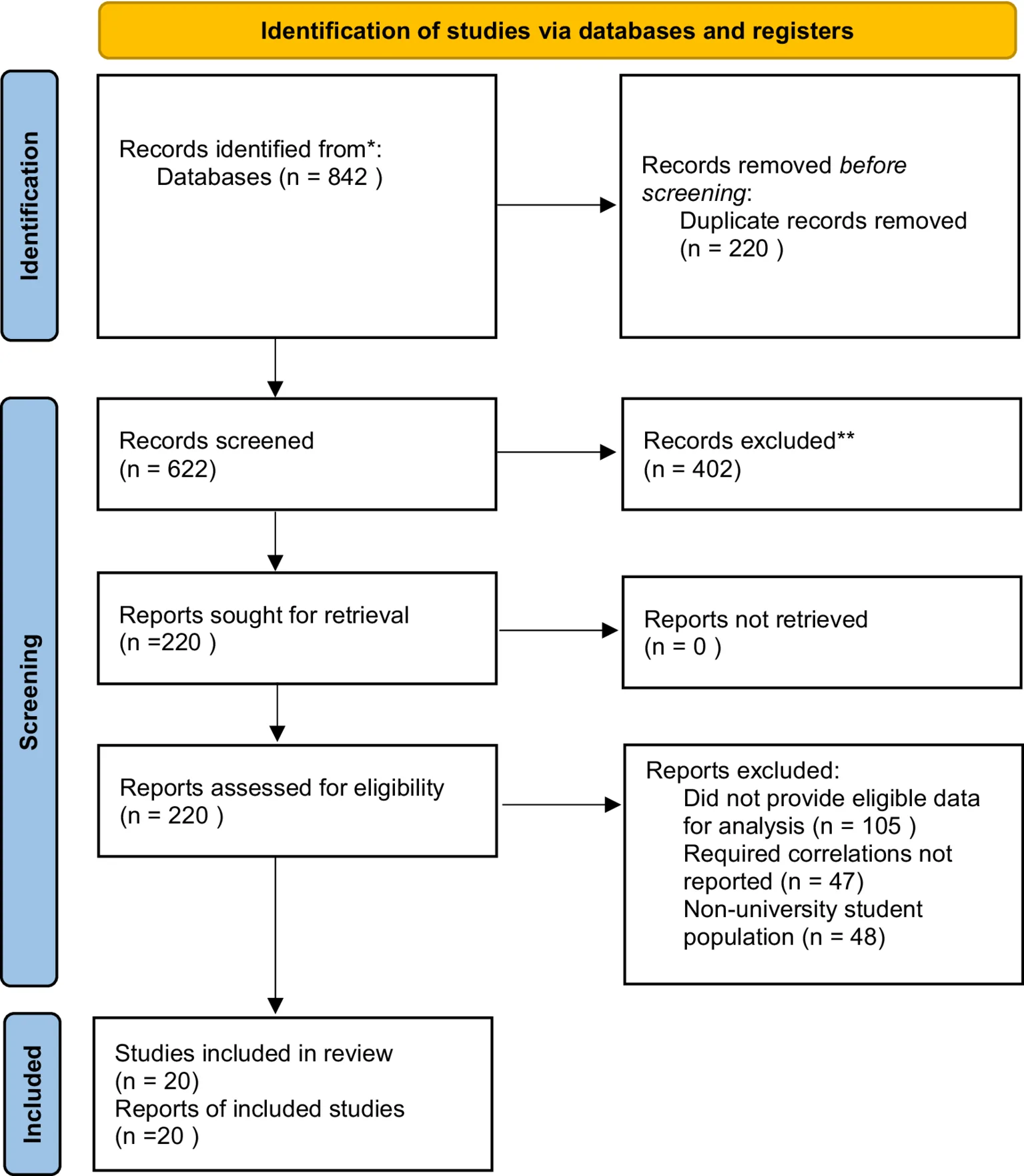
PRISMA 2020 flow of the data extraction process.

A summary of the characteristics of the 20 studies included in the analysis is displayed in Table 1. It summarizes sample characteristics, variables, statistical information extracted from each study.

**Table 1:** Characteristics of the studies.

Study	Sample	Variables examined	Statistical information
Peck (2017)	254 college students	Hope, academic self-efficacy, sense of belonging, persistence	Correlation and regression analysis
Thomas Jr et al. (2014)	139 college students	Self-efficacy, sense of belonging, campus involvement, persistence	Regression analysis
Jung and Lee (2018)	306 MOOC students	Academic self-efficacy, teaching presence, engagement, persistence	Structural equation modeling
Alamri (2022)	540 MOOC students	Academic self-efficacy, engagement, persistence, TAM variables	Structural equation modeling
Tovar (2013)	2,088 college students	Sense of belonging, engagement, mattering, persistence	Structural equation modeling
Zumbrunn et al. (2014)	212 undergraduates	Belonging, motivation, engagement, achievement, self-efficacy	Mixed methods
Wilson et al. (2015)	1,500 STEM undergraduates	Belonging, self-efficacy, emotional and behavioral engagement	Regression analysis
Vayre and Vonthron (2017)	255 online university learners	Social support, sense of community, self-efficacy, engagement	Path analysis
Vayre and Vonthron (2019)	506 online university learners	Social support, belonging, self-efficacy, engagement, achievement	Structural equation modeling
Handagoon and Varma (2019)	267 undergraduates	Social support, self-efficacy, belonging, psychological distress, engagement	Path Analysis
Painter (2012)	118 STEM undergraduates	Academic self-efficacy, sense of belonging, engagement	Regression analysis
Boyd et al. (2022)	408 undergraduates	Sense of belonging, engagement, GPA, retention	Structural equation Modeling
Han et al. (2017)	1,400 college students	Academic self-efficacy, sense of belonging, academic motivation, and persistence	Hierarchical cluster analysis
Banchefsky et al. (2019)	516 pSTEM undergraduates	Belonging, self-efficacy, persistence intentions	Path analysis
Lee and Song (2022)	277 MOOC students	Self-efficacy, task value, engagement, persistence	Structural equation modeling
Okolie et al. (2021)	599 undergraduates	Perceived supervisor support, belonging, engagement, self-efficacy, career curiosity	Path analysis
Lewis et al. (2017)	416 pSTEM undergraduates	Belonging, self-efficacy, persistence	Structural equation modeling
Chatman (2020)	1,276 undergraduates	Engagement, self-efficacy, belonging, GPA, persistence	Structural equation modeling
Adeshola and Agoyi (2023)	274 online university students	E-learning engagement, persistence, self-efficacy	Structural equation modeling
Yi et al. (2024)	308 postgraduate students	Belonging, academic self-efficacy, hardiness, engagement	Structural equation modeling

As shown in Table 1, the included studies exhibit substantial heterogeneity in context, sample characteristics, and construct operationalization. Several studies were conducted in online or MOOC settings, where participation is voluntary and dropout rates may be higher. Accordingly, variation in effect sizes may reflect not only theoretical differences but also disparities in learning environments, participation structures, and measurement approaches. Thus, a random-effects model was employed, assuming that true effect sizes vary across study contexts.

### 4.3 Coding

I prepared a coding table to systematically gather and arrange key information from each study. Effect sizes (represented as ‘*r*’) were extracted from the correlation matrices of each study. If the studies only provided standardized regression coefficients, *I* used Peterson and Brown (2005)‘s formula (*r* = *β* + 0.05λ, where *λ* = 1 when *β* ≥ 0 and *λ* = 0 when *β* < 0). When a correlation coefficient or an eligible standardized regression coefficient was not reported for a particular pair of variables, the corresponding cell was coded as missing (NA). Missing correlations were not assigned a value of zero and were not treated as observed effect sizes. The treatment of these incomplete correlation matrices during Stage 1 estimation is described below.

### 4.4 Data analysis

In Stage 1, the available correlation matrices were synthesized simultaneously using a random-effects model (Cheung, 2015a, 2015b). It was selected because heterogeneity in the population correlations was expected across studies due to differences in educational contexts, samples, and measurement instruments. Given the incomplete correlation structure across the primary studies, each study contributed information only for the correlations available in that study, while unavailable correlations remained missing effect sizes during estimation. Thus, a complete correlation matrix from every individual study was not required for inclusion in the Stage 1 synthesis. The six pooled correlations were estimated jointly within the random-effects Stage 1 model. A diagonal random-effects covariance structure was specified, and the weighted asymptotic covariance option was used for the sampling variance–covariance structure. In Stage 2, the pooled correlation matrix and its associated asymptotic covariance matrix were used as input for the weighted least-squares estimation of the hypothesized structural models (Jak et al., 2021). The complete asymptotic covariance matrix of the Stage 1 pooled correlations is reported in Supplementary Table S1.

The complete correlation matrix available in the dataset was checked for positive definiteness and was found to be positive definite; therefore, no correction or exclusion was required on this basis. Incomplete correlation matrices were retained and handled within the Stage 1 procedure as described above. For descriptive purposes, separate univariate random-effects meta-analyses were conducted for each pairwise association to estimate weighted correlations (*r*), 95% confidence intervals, and heterogeneity (*I*^2^), as reported in Table 2. The evaluation of correlation effect sizes was determined using Funder and Ozer’s (2019) guidelines.

**Table 2:** Summary of random-effects meta-analyses of pairwise correlations.

Association	*k*	Total sample	Pooled *r*	95% CI	***τ*** ^***2***^	*Q*	df	*p*	*I* ^2^	Egger’s *p*
per–bel	10	7,309	0.237	[0.117, 0.349]	0.038	223.468	9	<0.001	95.2%	0.33
per–ase	12	6,022	0.313	[0.194, 0.424]	0.047	340.121	11	<0.001	95.7%	0.54
per–enga	7	4,399	0.396	[0.224, 0.547]	0.065	175.783	6	<0.001	96.0%	–
enga–bel	10	6,683	0.239	[0.137, 0.335]	0.026	152.222	9	<0.001	95.4%	0.56
bel–ase	14	7,766	0.316	[0.223, 0.404]	0.033	215.984	13	<0.001	94.1%	0.91
enga–ase	11	4,622	0.402	[0.212, 0.556]	0.113	398.133	10	<0.001	95.8%	0.84

ase, academic self-efficacy, bel, sense of belonging, enga, engagement, per, persistence.

*k*, number of studies; *N*, total sample size; CI, confidence interval; τ^2^, between-study variance; *Q*, Cochran’s heterogeneity statistic; *I*^2^, percentage of total variability attributable to between-study heterogeneity. Correlations were synthesized using random-effects models with REML estimation after Fisher’s *z* transformation. The 95% confidence intervals were calculated on the Fisher’s **z** scale using the normal approximation and subsequently back-transformed to the correlation (*r*) metric. τ^2^ is reported on the Fisher’s *z* scale. Egger’s test was not conducted when *k* < 10.

I created both asymmetric and symmetric matrices using the metaSEM package as outlined by Cheung (2015b). An asymmetric matrix consists of coefficients that indicate one-way arrows connecting independent variables to dependent variables. A symmetric matrix shows coefficients that correspond to bidirectional relationships, indicating the variances within variables and the covariance across variables (Kanadlı et al., 2023). Model fit was evaluated using the chi-square (*χ*^2^) test, comparative fit index (CFI), Tucker–Lewis index (TLI), root mean square error of approximation (RMSEA), and standardized root mean square residual (SRMR). To determine statistical significance, 95% confidence intervals (CIs) were estimated. For the pooled correlations, 95% confidence intervals were calculated on the Fisher’s *z* scale using the normal approximation and subsequently back-transformed to the correlation (*r*) metric. For the structural path coefficients, asymptotic 95% confidence intervals based on the *z* approximation were reported, whereas likelihood-based 95% confidence intervals were used for the indirect effects. No bootstrap confidence intervals were used.

For moderator analysis, webMASEM (Jak et al., 2021) was used with learning environment coded as 0 for online and 1 for face-to-face. The moderator analysis included *k* = 7 studies in the online group and *k* = 13 studies in the face-to-face group. Moderation was evaluated using the omnibus test and the statistical significance of the moderation coefficients for the individual structural paths. Because the total number of included studies was limited (*k* = 20), moderator analysis was restricted to learning environment. Other potential moderators were not consistently available across the primary studies and therefore could not be systematically analyzed. Given the small subgroup sizes, subgroup-specific leave-one-study-out (LOSO) sensitivity analyses were also conducted. Parameter stability was evaluated based on the range and direction of the path coefficients across successfully estimated iterations.

## 5 Results

Prior to the TSSEM analysis, separate univariate random-effects meta-analyses were conducted for each pairwise correlation among sense of belonging, academic self-efficacy, engagement, and persistence. This analysis provided weighted correlations (*r*), 95% confidence intervals, and heterogeneity estimates (*I*^2^) for each association as detailed in Table 2.

As shown in Table 2, the *I*^2^ values ranged from 94.1 to 96.0%, indicating substantial between- study heterogeneity. The substantial heterogeneity may reflect the differences in study design (Gılıç et al., 2024), sample size, disciplinary fields, measurement instruments, and other contextual factors. Nevertheless, because of the limited number of studies reporting comparable variables, only the learning environment could be examined as a moderator in the present analysis. The results of Egger’s regression test for publication bias did not indicate significant funnel plot asymmetry. Funnel plots were also inspected and showed no substantial asymmetry. For engagement–persistence (*k* = 7), Egger’s test was not applied, as it is unreliable with fewer than 10 studies (Sterne and Egger, 2001). However, these findings should be interpreted cautiously given the relatively small number of studies contributing to several associations.

Following the separate univariate meta-analyses, the available correlations were jointly synthesized using the random-effects Stage 1 procedure of TSSEM, accounting for sampling dependencies and the incomplete correlation structure across studies. The pooled correlations were *r* = 0.237 for persistence–belonging, *r* = 0.313 for persistence–academic self-efficacy, *r* = 0.396 for persistence–engagement, *r* = 0.239 for engagement–belonging, *r* = 0.316 for belonging–academic self-efficacy, and *r* = 0.402 for engagement–academic self-efficacy. The pooled correlation matrix and its asymptotic covariance matrix were subsequently used as input for the Stage 2 structural analysis.

The partial mediation model showed mixed but generally acceptable fit indices, *χ*^2^ = 8.44, *p* > 0.05, CFI = 0.92, TLI = 0.83, RMSEA = 0.02, 95% CI [0.011, 0.031], SRMR = 0.09, AIC = −14.13, BIC = 53.64. Although TLI fell below the ideal threshold, this may reflect its sensitivity to sample size (Kenny and McCoach, 2003). A visual representation of the partial mediation model is shown in Figure 4.

**Figure 4:**
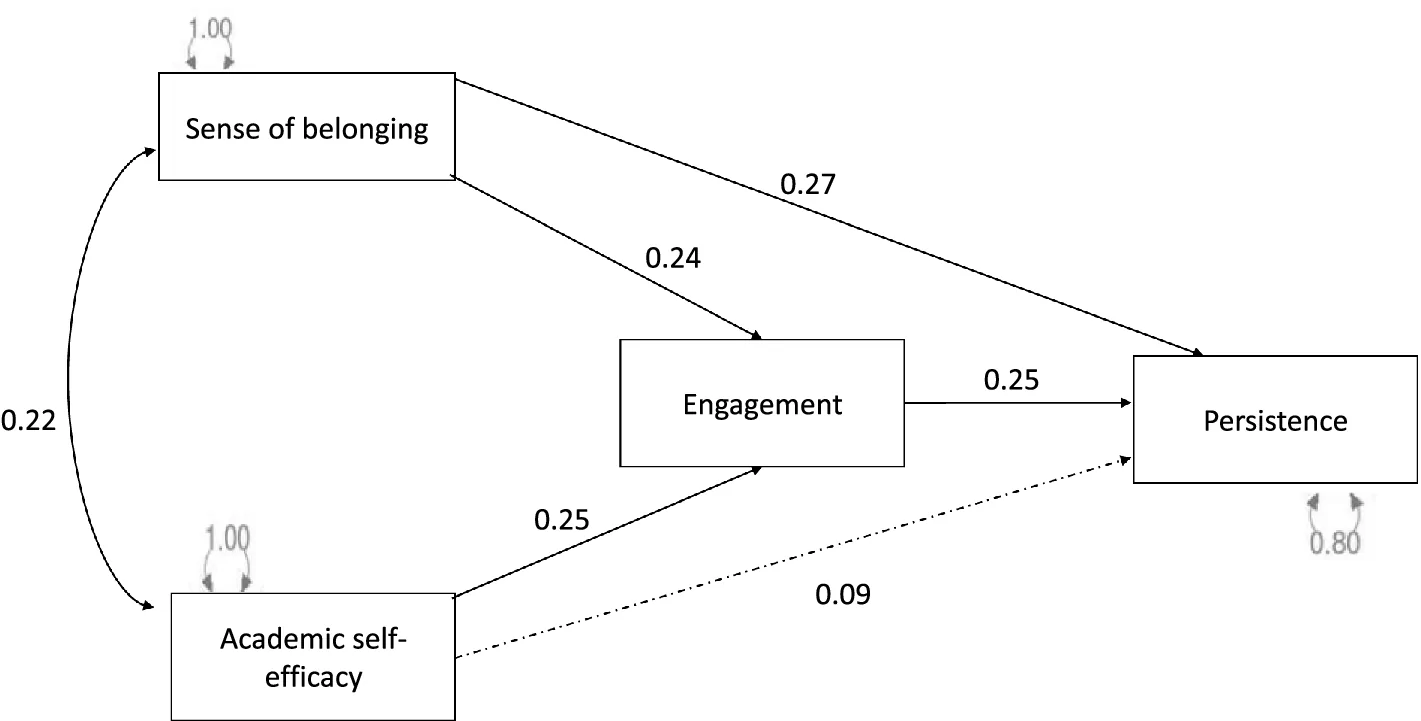
The partial mediation model.

Sense of belonging was significantly associated with engagement (*β* = 0.241, 95% CI [0.122, 0.358]), and academic self-efficacy was also significantly associated with engagement (*β* = 0.255, 95% CI [0.158, 0.348]). Engagement, in turn, was significantly associated with persistence (*β* = 0.259, 95% CI [0.076, 0.428]). Regarding direct associations, sense of belonging was significantly associated with persistence (*β* = 0.276, 95% CI [0.099, 0.447]), whereas the direct path from academic self-efficacy to persistence was not statistically significant (*β* = 0.097, 95% CI [−0.025, 0.210]). The indirect association via engagement was significant (0.061, 95% likelihood-based CI [0.020, 0.120]), indicating a significant indirect pathway through engagement. These findings suggest that engagement partially mediates the association between sense of belonging and persistence, while academic self-efficacy is primarily associated with persistence through engagement. Therefore, H1 was partially supported. Sense of belonging showed both a significant direct association with persistence and a significant indirect association through engagement. In contrast, academic self-efficacy was indirectly associated with persistence through engagement, whereas its hypothesized direct association with persistence was not statistically significant.

Given the relatively small number of studies, a leave-one-study-out (LOSO) sensitivity analysis was conducted to assess parameter stability. All models were successfully estimated, and all structural coefficients retained their positive direction across iterations, indicating that the structural estimates were reasonably stable to the omission of individual studies (see Supplementary Table S2).

As an exploratory analysis, an alternative partial mediation model was also examined. This alternative specification was not part of the *a priori* hypothesized model and is therefore presented as a post-hoc exploratory analysis rather than as confirmatory evidence. The direct path from academic self-efficacy to persistence was found to be non-significant in the initial model. This finding is consistent with prior research suggesting that academic self-efficacy primarily influences persistence indirectly through engagement rather than exerting a direct effect (Jung and Lee, 2018; Kahu and Nelson, 2018). Based on both theoretical considerations and empirical results, the direct path from academic self-efficacy to persistence was removed, resulting in a revised partial mediation model. In this specification, engagement serves as the primary mediating mechanism linking academic self-efficacy to persistence. In contrast, sense of belonging, as an indicator of students’ social integration, was retained as both a direct and indirect predictor of persistence, in line with theoretical frameworks emphasizing its dual role (Tinto, 1993; Gopalan and Brady, 2020). The revised model is presented in Figure 5.

**Figure 5:**
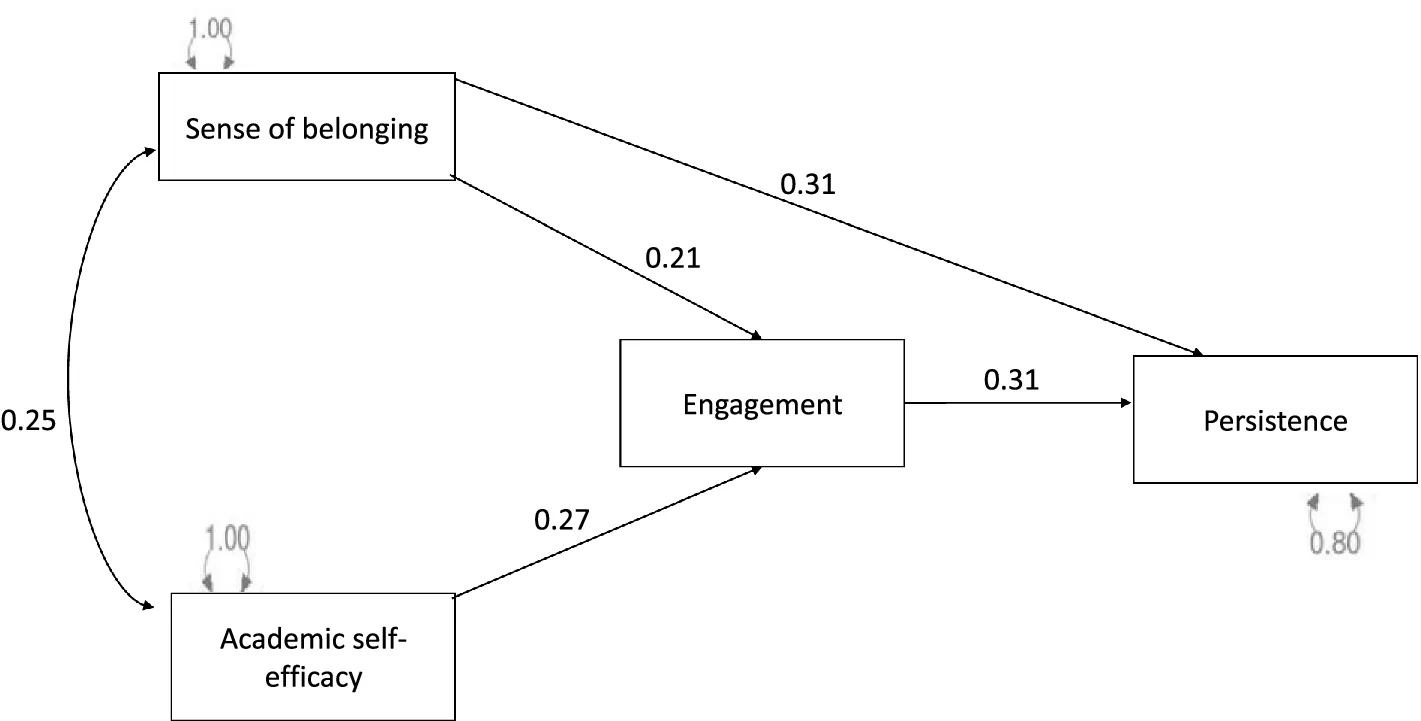
The revised partial mediation model.

The revised partial mediation model demonstrated good fit to the data (*χ*^2^ = 3.49, *p* > 0.05, CFI = 0.99, TLI = 0.95, RMSEA = 0.01, 95% CI [0.000, 0.027], SRMR = 0.03, AIC = 0.49, BIC = −7.11). Sense of belonging was significantly associated with engagement (*β* = 0.219, 95% CI [0.102, 0.336]), and academic self-efficacy was also significantly associated with engagement (*β* = 0.270, 95% CI [0.175, 0.365]). Engagement, in turn, was significantly associated with persistence (*β* = 0.317, 95% CI [0.161, 0.474]). Regarding direct associations, sense of belonging was positively and significantly associated with persistence (*β* = 0.311, 95% CI [0.143, 0.480]). In addition, sense of belonging and academic self-efficacy were positively correlated (r = 0.254, 95% CI [0.153, 0.356]). The indirect associations via engagement were positive and statistically significant for both predictors (sense of belonging: 0.069, 95% likelihood-based CI [0.032, 0.121]; academic self-efficacy: 0.086, 95% likelihood-based CI [0.046, 0.146]), indicating the mediating role of engagement.

A full mediation model was also tested by constraining the direct paths from sense of belonging and academic self-efficacy to persistence to zero as shown in Figure 6.

**Figure 6:**
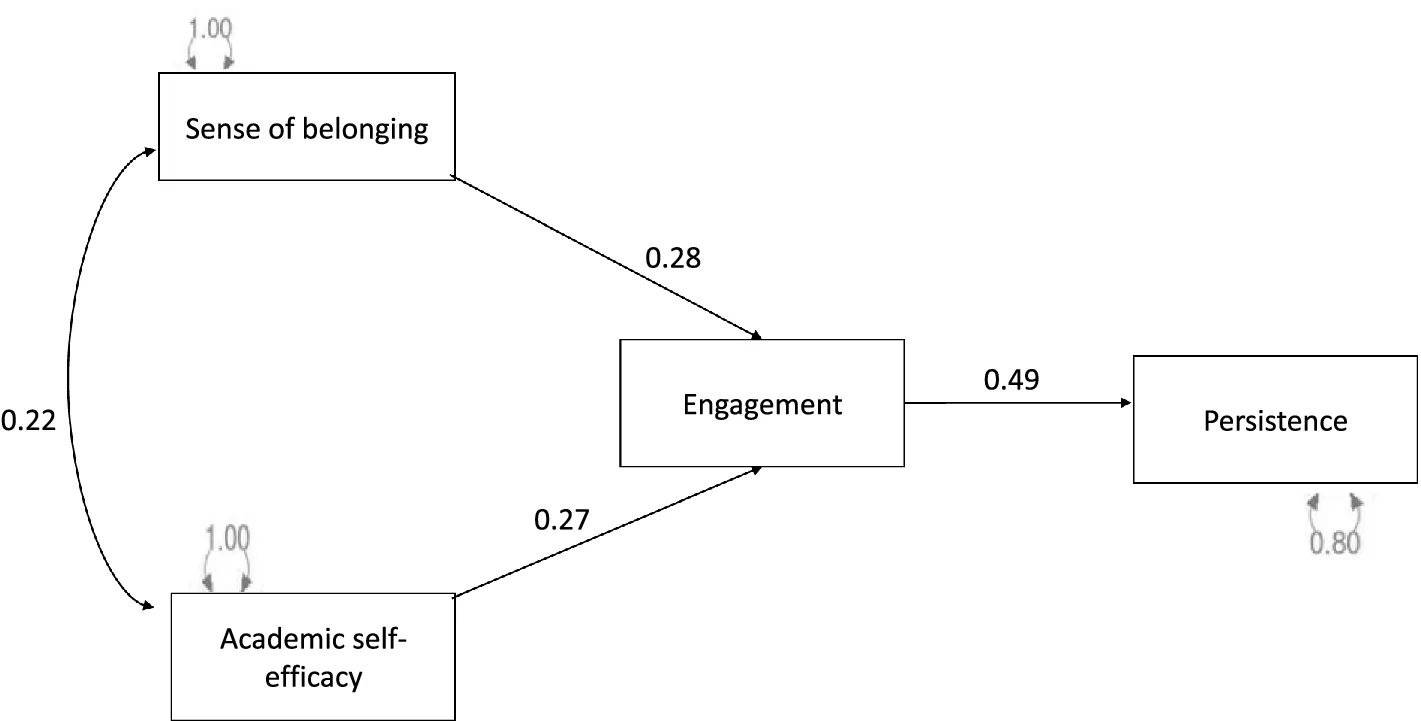
Full mediation model.

A full mediation model showed less favourable fit indices, *χ*^2^ = 14.23, *p* > 0.05, CFI = 0.93, TLI = 0.78, RMSEA = 0.02, SRMR = 0.10, AIC = 10.23, BIC = −4.97, than the revised partial mediation model. Sense of belonging was significantly associated with engagement (*β* = 0.288, 95% CI [0.175, 0.398]), and academic self-efficacy was also significantly associated with engagement (*β* = 0.276, 95% CI [0.186, 0.364]). Engagement, in turn, was significantly associated with persistence (*β* = 0.493, 95% CI [0.373, 0.613]). The indirect association via engagement was positive and statistically significant (0.142, 95% likelihood-based CI [0.081, 0.213]), indicating a mediating role of engagement in the relationship between the predictors and persistence.

The full mediation model showed less favorable fit indices than the revised partial mediation specification. However, because the revised model was examined *post hoc*, these descriptive differences should not be interpreted as confirmatory evidence of the superiority of the revised model. Overall, the pattern of results did not provide support for the hypothesized full mediation structure; therefore, H2 was not supported. No conventional chi-square difference test was used to infer statistically superior model fit because the models were estimated from meta-analytically synthesized correlations within the two-stage TSSEM framework.

### 5.1 Moderation

Approximately 65% of the studies were conducted in face-to-face settings. The omnibus test of moderation was significant at α = 0.05, with *χ*^2^(4) = 28.51, *p* < 0.001, indicating that at least one structural path was moderated by the learning environment, providing partial support for H3. The results showed that the association between sense of belonging and engagement was significantly moderated by the learning environment (*β* = 0.150, *p* = 0.002), as was the direct path linking sense of belonging to persistence (*β* = 0.136, *p* = 0.001). In contrast, the moderating effects for the path from academic self-efficacy to engagement (*β* = −0.047, *p* = 0.426), and from engagement to persistence (*β* = 0.040, *p* = 0.582) were not statistically significant. Thus, the results suggest that associations involving sense of belonging may vary across learning environments.

Subgroup-specific leave-one-study-out (LOSO) analyses were conducted to assess parameter stability. All 13 face-to-face iterations were successfully estimated, with all structural paths retaining their direction. In the online subgroup, six of seven iterations were estimable, and three of four paths retained their direction. The sense of belonging → engagement coefficient was sensitive to study omission (*β* = −0.162 to 0.151), whereas the other examined paths retained their direction. Thus, the moderation finding for the sense of belonging → engagement pathway should be interpreted cautiously (see Supplementary Table S3).

## 6 Discussion

### 6.1 The role of engagement in persistence

This study examined the associations among sense of belonging, academic self-efficacy, engagement, and persistence using a MASEM. The findings indicate that engagement is associated with students’ persistence. Higher levels of engagement were associated with higher levels of persistence, consistent with previous findings linking engagement to persistence (Alamri, 2022; Lee and Song, 2022). The pooled correlations suggest that engagement was more strongly associated with persistence than academic self-efficacy and sense of belonging. This pattern is consistent with theoretical accounts that conceptualize engagement as a proximal mechanism linking students’ psychological and social experiences with persistence-related outcomes (Kahu and Nelson, 2018). From this perspective, engagement can be understood as one of the most immediate manifestations of students’ involvement in their academic environment and may be closely associated with their likelihood of continuing their studies (Fredricks et al., 2004).

The substantial heterogeneity across studies indicates that the strength of these relationships varies across contexts, potentially reflecting differences in measurement approaches, institutional settings, and student populations. One possible explanation is that engagement is more proximal to persistence than the psychological and social factors that precede it. By reflecting students’ active participation, effort, and involvement in academic activities, engagement may represent the behavioral process through which students maintain their connection to their studies (Fredricks et al., 2004; Kahu and Nelson, 2018). Thus, contextual differences may alter the strength of this association without necessarily eliminating the underlying link between academic involvement and persistence (Tinto, 1993, 2017). This may explain why individual studies have reported associations of varying magnitude while the meta-analytic synthesis still revealed an overall positive relationship.

### 6.2 Comparison of model specifications

The partial and full mediation models were specified *a priori* to test H1 and H2, respectively. In addition to these hypothesized models, a revised partial mediation model was examined following the evaluation of the partial mediation model. Because this revised specification was not specified *a priori*, it should be interpreted as a post-hoc exploratory model rather than as a confirmatory test of the proposed theoretical pathways.

Although the partial mediation model specified both direct and indirect pathways from academic self-efficacy, the non-significant direct path suggests that its association with persistence is primarily indirect. Variation in the extent to which learning contexts enable this translation from beliefs into engagement may partly explain the inconsistent self-efficacy–persistence associations reported across studies (Jung and Lee, 2018; Kahu and Nelson, 2018).

Sense of belonging showed both direct and indirect associations with persistence in the revised partial model. This dual pathway may reflect the broader role of belonging in persistence: sense of belonging may encourage engagement while also strengthening students’ commitment to remain enrolled (Tinto, 1993; Gopalan and Brady, 2020). These findings are consistent with Tinto’s (1993) integration model, which positions social integration as a critical element in students’ persistence. Research also suggests that sense of belonging not only fosters engagement but may also be independently associated with students’ commitment to persist (Gopalan and Brady, 2020; Murphy et al., 2020). However, because this model was examined *post hoc*, the findings should be interpreted as exploratory rather than as confirmation of a particular mediation structure.

The full mediation model showed descriptively less favorable fit indices than the revised partial mediation specification. Although this pattern may suggest that a fully mediated structure provides a less adequate representation of the observed associations, the comparison should be interpreted cautiously given the exploratory status of the revised specification. Importantly, the full mediation model still highlighted the central role of engagement in persistence, consistent with prior research (Pascarella and Terenzini, 2005; Kahu, 2013; Tinto, 2017).

These findings are also consistent with Social Cognitive Theory (Bandura, 1997), which suggests that personal beliefs influence behavior and, in turn, shape outcomes. Academic self-efficacy and sense of belonging may represent important psychological and social factors associated with engagement. In particular, the indirect path from academic self-efficacy to persistence through engagement aligns with the SCT proposition that personal beliefs primarily influence outcomes through behavioral mechanisms such as effort and participation (Schunk and DiBenedetto, 2020). However, the finding that sense of belonging retained a direct association with persistence, in addition to its indirect pathway through engagement, suggests that persistence may involve processes extending beyond those represented by engagement alone.

### 6.3 Moderating role of learning environment

The moderation findings provide one possible explanation for the heterogeneity observed across studies. The associations of sense of belonging with engagement and persistence varied across learning environments, suggesting that the role of sense of belonging may depend partly on the opportunities for social interaction and integration provided by the learning context. In line with SCT, the significant moderation effects observed for the paths involving sense of belonging suggest that environmental factors may be not only antecedents but also conditions that influence the strength of relationships within the model. Consistent with prior research, face-to-face environments may provide greater opportunities for social interaction and the development of sense of belonging (Farrell and Brunton, 2020; Strayhorn, 2019). The absence of rich communication channels may weaken its association with engagement. These findings highlight the importance of designing online learning environments that foster interaction, community, and instructor presence (Farrell and Brunton, 2020; Vo and Ho, 2024). However, the subgroup-specific LOSO analysis showed that the sense of belonging–engagement pathway was sensitive to the omission of individual studies in the online subgroup. Therefore, the moderation finding for this pathway should be interpreted cautiously.

In contrast, learning environment did not significantly moderate the associations between academic self-efficacy and engagement or between engagement and persistence. While prior research suggests that self-efficacy may play a stronger role in online settings (Zimmerman and Kulikowich, 2016), the present findings provided no evidence that its association with engagement differed significantly across learning environments. Similarly, no significant difference across learning environments was observed for the association between engagement and persistence. This finding is consistent with previous evidence suggesting that the relationship between engagement and persistence may operate similarly across instructional modes (Zeng and Xin, 2025; Yang et al., 2024).

### 6.4 Limitations and future directions

Engagement is a multifaceted construct that is conceptualized and operationalized differently across studies, posing challenges for meta-analytic synthesis. Engagement is typically defined as behavioral, emotional and cognitive components (Jung and Lee, 2018; Vayre and Vonthron, 2019). Persistence is also operationalized either as students’ intentions to continue their studies or as behavioral outcomes such as actual retention or dropout. This diversity increases the risk of an “apples and oranges” problem, as pooling heterogeneous indicators may obscure important distinctions between constructs. Ideally, these differences would be examined through subgroup analyses. However, the number of studies contributing to the individual pairwise associations in the present meta-analysis was limited. Further subdivision according to specific operationalizations of engagement and persistence would have resulted in very small and uneven subgroups, limiting the stability and interpretability of moderator estimates. Therefore, operationalization-specific subgroup analyses were not conducted. Thus, the findings should not be taken to imply that behavioral, cognitive, and emotional engagement contribute equally to persistence, or that associations with intentions to persist necessarily translate into actual retention. Future meta-analytic research with a larger evidence base should examine these operationalizations separately.

Furthermore, the included studies varied substantially in their research contexts, including differences between MOOC environments and traditional university settings. Such variation may contribute to the substantial between-study heterogeneity observed in the pooled associations. Although learning environment was examined as a moderator, other potentially relevant moderators could not be systematically evaluated because they were not consistently reported across primary studies. Moreover, learning environment was coded at the study level, which may not fully capture variation in students’ actual learning experiences within individual studies. Future meta-analytic research with a larger and more consistently documented evidence base should examine these sources of heterogeneity.

The correlation coefficients across the research indicated that only the study conducted by Vayre and Vonthron (2019) provided all correlation coefficients for the variables. Thus, the analysis relied substantially on incomplete correlation matrices, with different subsets of studies contributing information to different associations. Although TSSEM accommodates such incomplete correlation structures, this limits the amount of information available for estimating the complete model. Consequently, the lack of studies limits the generalizability of the results. This limitation is particularly relevant to the moderator analysis, where the online subgroup contained only seven studies. The moderation findings should therefore be interpreted cautiously and require replication as the evidence base expands. To minimize potential dependence among samples, studies identified as using the same or overlapping samples were excluded during study selection. Nevertheless, unreported sample overlap across independent publications cannot be completely ruled out. Moreover, many of the included studies relied on cross-sectional rather than longitudinal data, highlighting the need for additional longitudinal research examining the proposed model.

Although publication bias was assessed using Egger’s regression test, it should be interpreted cautiously because several associations were based on a relatively small number of studies. In particular, Egger’s test was not conducted for the engagement–persistence association (*k* = 7), given the limited reliability of regression-based asymmetry tests with small numbers of studies. In addition, the search was restricted to English-language studies and to the selected databases and Google Scholar, potentially introducing language and database coverage bias. Although Google Scholar broadened the scope of the search, differences in its indexing and search functionality may also affect search reproducibility. The conversion of standardized regression coefficients (β) to correlations (*r*) when correlations were unavailable may have introduced some approximation error into the synthesized estimates.

The meta-analytic design relies largely on observational data, which limits the ability to establish causal relationships among the variables, particularly in the presence of potential unmeasured confounding. In addition, the revised partial mediation model was specified following evaluation of the initially hypothesized model and should therefore be considered post-hoc and exploratory rather than confirmatory. While academic performance is widely recognized as a strong predictor of persistence, the present study focuses specifically on psychological mechanisms rather than performance outcomes. Accordingly, the reported indirect effects should be interpreted as statistical associations rather than evidence of causal pathways. Future research should integrate performance indicators within more comprehensive models.

## Data Availability

The raw data supporting the conclusions of this article will be made available by the authors, without undue reservation.
