# Supplementary material for: Associations of sense of belonging and academic self-efficacy with college persistence through student engagement: MASEM with moderation by learning environment
Source: Front Psychol. 2026 Sep 30;17:1858552. doi: 10.3389/fpsyg.2026.1858552 (PMC13662114; doi:10.3389/fpsyg.2026.1858552)
Supplement: Supplementary file 1 [file Data_Sheet_1.pdf]

Table S1

Asymptotic Covariance Matrix of the Stage 1 Pooled Correlations

Association	per–bel	per–ase	per–enga	enga–bel	bel–ase	enga–ase
per–bel	.002927	.000044	.000014	.000005	.000021	.000003
per–ase	.000044	.003021	.000048	.000003	.000020	.000023
per–enga	.000014	.000048	.005704	.000011	.000001	.000026
enga–bel	.000005	.000003	.000011	.001981	.000035	.000040
bel–ase	.000021	.000020	.000001	.000035	.001865	.000021
enga–ase	.000003	.000023	.000026	.000040	.000021	.005143

Note. per = persistence; bel = sense of belonging; ase = academic self-efficacy; enga = engagement.

Table S2

Leave-One-Study-Out Sensitivity Analysis for the Partial Mediation Model

Structural path	Full-model estimate	Minimum LOSO estimate	Maximum LOSO estimate	Direction retained
Belonging → Engagement	.241	.109	.163	Yes
Academic self-efficacy → Engagement	.255	.274	.356	Yes
Belonging → Persistence	.276	.076	.133	Yes
Academic self-efficacy → Persistence	.097	.136	.196	Yes
Engagement → Persistence	.259	.248	.349	Yes

Note. LOSO = leave-one-study-out.

Table S3

Leave-One-Study-Out Sensitivity Analysis of Structural Paths by Learning Environment

**Learning environment**	**Structural path**	**Minimum LOSO estimate**	**Maximum LOSO estimate**	**Direction retained**
Online	Belonging → Engagement	−.162	.151	No
	Self-efficacy → Engagement	.632	.730	Yes
	Belonging → Persistence	.199	.335	Yes
	Engagement → Persistence	.452	.518	Yes
Face-to-face	Belonging → Engagement	.072	.144	Yes
	Self-efficacy → Engagement	.122	.283	Yes
	Belonging → Persistence	.159	.268	Yes
	Engagement → Persistence	.043	.181	Yes

Note. LOSO = leave-one-study-out.
